# Clinical Characteristics of Tick-Borne Encephalitis in Adult Patients: A 10-year Retrospective Study in Stockholm, Sweden

**DOI:** 10.1093/infdis/jiae463

**Published:** 2024-09-24

**Authors:** Sofia Bartholdsson, Maria-Pia Hergens, Karin E Hansson, Josef Ragnarsson, Peter Hodosi, Ismail Kus, Mona Insulander, Sirkka Vene, Lars Lindquist, Helena H Askling, Sara Gredmark-Russ

**Affiliations:** Center for Infectious Medicine, Department of Medicine Huddinge, Karolinska Institutet, Stockholm, Sweden; Department of Infectious Diseases, Karolinska University Hospital, Stockholm, Sweden; Division of Infectious Diseases, Department of Medicine Solna, Karolinska Institutet, Stockholm, Sweden; Department of Communicable Disease Control and Prevention, Region Stockholm, Stockholm, Sweden; Department of Infectious Diseases, Södersjukhuset Stockholm, Sweden; Department of Infectious Diseases, University Hospital of Umeå, Umeå, Sweden; Center for Infectious Medicine, Department of Medicine Huddinge, Karolinska Institutet, Stockholm, Sweden; Center for Infectious Medicine, Department of Medicine Huddinge, Karolinska Institutet, Stockholm, Sweden; Department of Communicable Disease Control and Prevention, Region Stockholm, Stockholm, Sweden; The Public Health Agency of Sweden, Solna, Sweden; Division of Infectious Diseases, Department of Medicine Solna, Karolinska Institutet, Stockholm, Sweden; Division of Infectious Diseases, Department of Medicine Solna, Karolinska Institutet, Stockholm, Sweden; Academic Specialist Centre, Stockholm Health Services, Region Stockholm, Stockholm, Sweden; Center for Infectious Medicine, Department of Medicine Huddinge, Karolinska Institutet, Stockholm, Sweden; Department of Infectious Diseases, Karolinska University Hospital, Stockholm, Sweden; Laboratory for Molecular Infection Medicine Sweden, Umeå, Sweden

**Keywords:** tick-borne encephalitis, severity, sequelae, healthcare utilization, Sweden

## Abstract

**Background:**

The incidence of tick-borne encephalitis (TBE) has increased during the last decades in Europe. Our aim was to assess the clinical characteristics and outcome of patients with TBE in Region Stockholm, as a high-risk area in Sweden.

**Methods:**

The notification database at the regional Department of Communicable Disease Control and Prevention was used to identify TBE cases during 2006–2015. Clinical data were retrieved from the included patients’ medical records. The associations of specific variables to predefined outcomes of disease severity were evaluated with multivariate logistic regression models.

**Results:**

Of 1004 identified TBE cases, 703 adult patients were included. Sixty-one percent were men, and the median age was 50 years (range, 18–94 years). The majority of patients were nonvaccinated. Comorbidity was present in 34%, and 4% were receiving immunomodulatory therapy. Seventy-five percent were hospitalized, and 11% had severe disease. More than 70% of the 79 patients followed up for >6 months had persisting symptoms. The case fatality rate was 1.4%, 15% in the group with immunomodulatory treatment. In the multivariate analysis, severe disease was associated with underlying comorbid conditions, age ≥50 years, and previous complete TBE vaccination.

**Conclusions:**

This is the largest cohort of patients with TBE in Scandinavia. Our findings of a more severe course of disease in older patients, those receiving immunomodulatory therapy, those with comorbid conditions, and those with vaccination breakthrough infections must be interpreted in the context of hospitalized patients. Optimized prevention is needed for patients receiving immunomodulatory therapy, given the considerable case fatality rate. Follow-up visits and rehabilitation should be better standardized.

## Teaser

Among 703 patients with tick-borne encephalitis in Sweden, a more severe course of disease was found in older patients and in those with immunomodulatory therapy, comorbid conditions, or vaccination breakthrough infection. Patients with immunomodulatory therapy had a considerable case fatality rate.

## Graphical Abstract

**Graphical Abstract:**
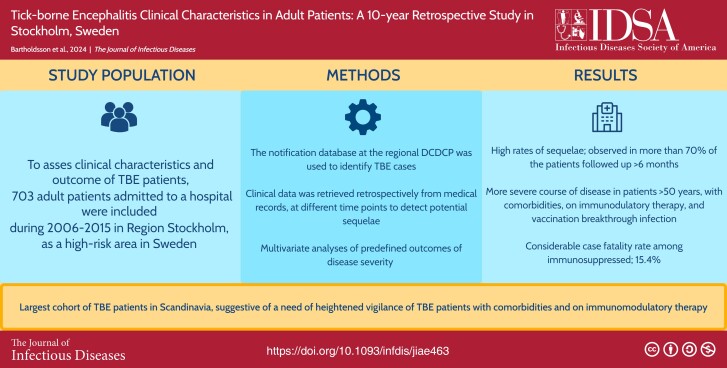
This graphical abstract is also available at Tidbit: https://tidbitapp.io/tidbits/tick-borne-encephalitis-clinical-characteristics-in-adult-patients-a-10-year-retrospective-study-in-stockholm-sweden?utm_campaign=tidbitlinkshare&utm_source=ITP

Tick-borne encephalitis (TBE) is a viral infectious disease involving the central nervous system and is predominantly transmitted to humans by infected hard ticks, mainly *Ixodes ricinus* and *Ixodes persulcatus* [1]. The infection is occasionally acquired by consumption of infected unpasteurized dairy products and more rarely by breastfeeding, blood transfusions, organ transplantation, or laboratory exposure [2–5].

TBE is a disease of varying severity, ranging from mild symptoms to severe encephalitis. It often presents with a biphasic course. The initial phase correlates with viremia and includes nonspecific symptoms, such as fever, headache, and myalgia [5, 6], and is followed by an asymptomatic period [2, 5–7]. In the second phase, meningitis or meningoencephalitis develops, while some patients present with spinal involvement (meningoencephalomyelitis) [2, 6]. The spectrum of neurological symptoms is broad [4]. Many patients are hospitalized, and some require intensive care, while the mortality rate is rather low with a case fatality rate of about 0.5%–2% [2, 4, 6–9]. At discharge from the hospital, many patients are still experiencing symptoms [7, 8], and 20%–50% report incomplete recovery [6, 10–12].

There is no available evidence-based treatment for TBE. However, there are highly effective, inactivated vaccines, with an estimated field effectiveness in adults of 96%–99% [13, 14]. TBE vaccination breakthrough cases are rare [5]. Increasing age has been suggested to be associated with an inferior vaccine response [15–17]. These findings, however, are subject to different definitions of breakthrough infections and to how and when immunizations were performed, and the immune status of the patients is often unknown.

Although TBE is preventable to a high extent by vaccination, the incidence is increasing and represents a growing public health problem [1, 5, 18]. This also applies to Sweden, with 300–600 current diagnoses each year. The average annual cost for TBE-related healthcare in Sweden for 2015–2019 was estimated at 24.5 million Euros [19].

Clinical data on TBE in the setting of Swedish healthcare are scarce [20–22]. Over the last years, several large cohort studies have been published from Europe, but none from high-endemic areas in the northern countries. The aim of the present study was to obtain detailed clinical characteristics of patients with TBE in Region Stockholm, with 2.1 million inhabitants, as one of the most highly endemic areas in Sweden, and to analyze potential risk factors for the development of severe TBE.

## PATIENTS AND METHODS

### Study Design

We performed a retrospective longitudinal study of patients with TBE in Region Stockholm. All notified TBE cases from 2006 to 2015 were identified through the national notification database of the regional Department of Communicable Disease Control and Prevention. TBE is a notifiable disease in Sweden, with mandatory reporting by the clinicians and microbiology laboratories, providing a high notification rate. Defined cases followed the national notification definition [23], in agreement with the criteria of the European Centre for Disease Control [24]. TBE was verified in all patients by using an enzyme-linked immunosorbent assay for detection of specific immunoglobulin (Ig) M and IgG antibodies in serum, performed by the hospitals’ laboratories. Additional enzyme-linked immunosorbent assays and/or neutralization tests were performed in patients with vaccination breakthrough infections, and these results have been previously published [17].

Previously vaccinated patients were categorized into previous vaccination according to the recommended schedule in Sweden during a specific year (described as “completely vaccinated” and thoroughly presented elsewhere [17]) and vaccination that did not adhere to the recommended vaccination schedule (“incompletely vaccinated”). The standard vaccination schedule was initially 2 primary doses (at 0 and 1–2 months), followed by additional doses at 5–12 months and 3 years, and further doses thereafter every 5 years. From 2010, an extra priming dose at month 3 was recommended for all individuals aged ≥60 years.

### Ethical Considerations

The study was approved by the Regional Ethical Review Board in Stockholm (Diary Number 2016/1902-31/4).

### Data Collection

Adult patients, ≥18 years of age, were included in the study. Data were previously collected, including vaccine doses regarding the completely vaccinated patients [17], and used in the present study. Herein, we included all the patients with TBE seeking healthcare at the 4 main hospitals in Region Stockholm (Karolinska University Hospital, Danderyds Hospital, Södersjukhuset, and St Göran Hospital), both inpatients and outpatients. Clinical and laboratory data were retrieved retrospectively from medical records. Data on symptoms and findings were collected at 4 predefined time points: 0 to <2 weeks, ≥2 weeks to <3 months, ≥3 to <6 months, and ≥6 to 12 months.

### Classification of Disease Severity

An assessment of the severity of the disease in the acute phase was performed, according to the definition used in earlier studies [25]. Patients were thereby classified as having mild, moderate, or severe TBE. Primarily meningeal symptoms, such as fever, headache, neck stiffness, and sensitivity to light and/or sound, were categorized as mild disease. Moderate signs of encephalitis, with or without altered consciousness, and/or diffuse or focal neurological symptoms were categorized as moderate disease. Multifocal symptoms and/or severe signs of encephalitis with altered consciousness were categorized as severe disease.

### Statistical Analysis

Differences in clinical characteristics were presented by vaccination status, underlying comorbid conditions, immunomodulatory treatment, and for 2 age groups (<50 or ≥50 years). Categorical variables were expressed as frequencies (percentages), and differences were determined by means of χ^2^ or Fisher exact tests. Continuous variables were expressed as medians and means, and differences in means were analyzed using *t* tests. *P* values <.05 indicate significant differences.

The relationships between age, sex, comorbidity, immunomodulatory treatment, previous TBE vaccination, and disease severity were measured as odds ratios through multivariate logistic regression models. The following categories were used as outcomes for disease severity: moderate and severe disease, hospitalization, hospitalization for >7 days, treatment in the ICU, assisted ventilation, and case fatality. SAS Enterprise software (SAS Institute) was used for all statistical analyses.

## RESULTS

### Patient Characteristics and Clinical Presentation

**Figure 1.**
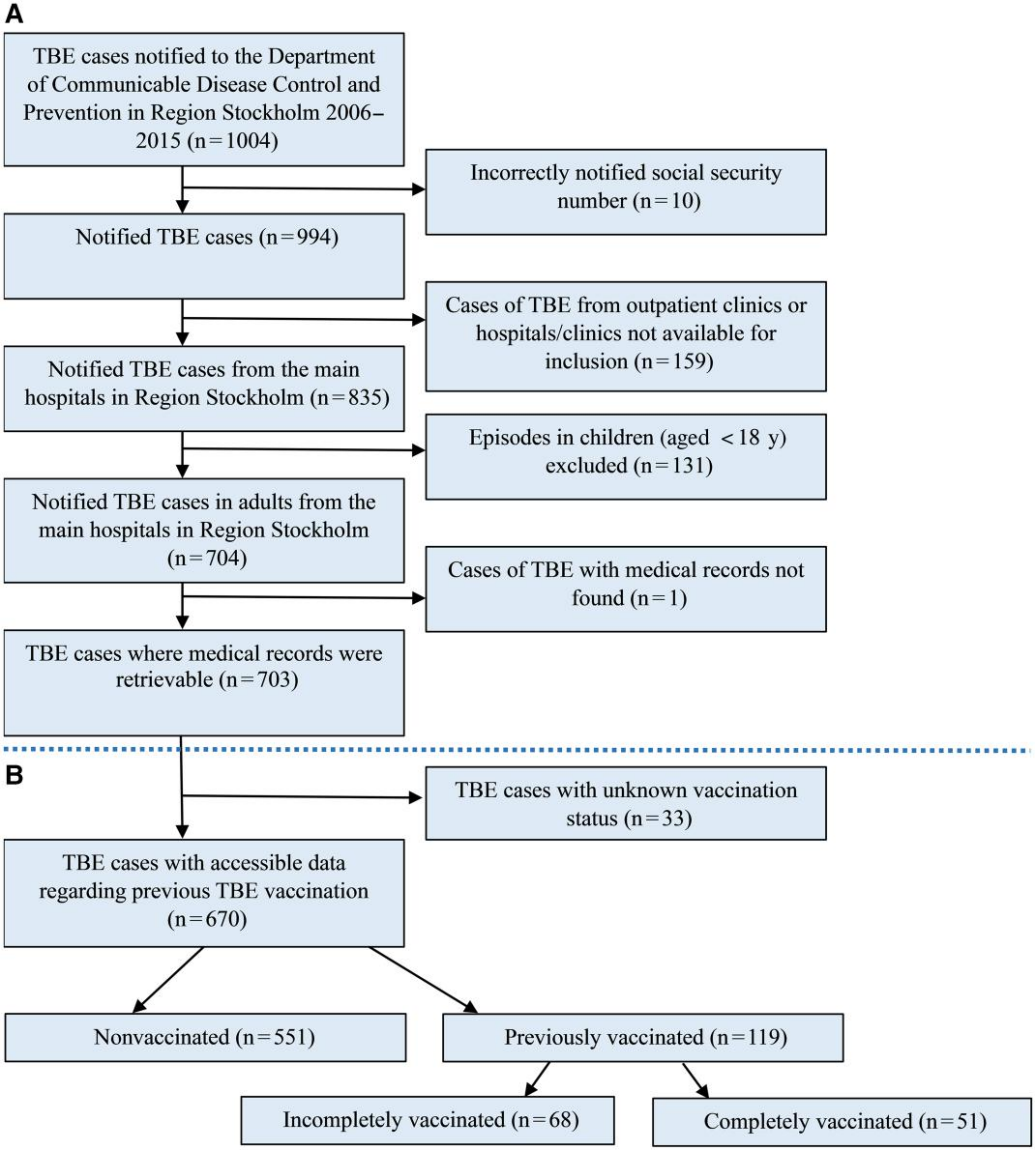
*A*, Flow chart of the included and excluded patients in the study. *B*, Accessible data regarding previous tick-borne encephalitis (TBE) vaccination, among included patients.

From 2006 to 2015, we retrieved data from a total of 703 patients, representing 81% (703 of 863) of notified adult TBE cases in Region Stockholm (Figure 1). Ninety-five percent of the included patients had accessible data regarding previous TBE vaccination, of whom 82% were nonvaccinated (Figure 1*B*). The patients’ characteristics and clinical presentations are presented in Table 1. Underlying comorbidity was present in 242 patients (34%), and 26 (4%) were on immunomodulatory therapy. The majority had mild or moderate disease. Seventy-five percent were hospitalized, and 6% required care within the ICU. The outcome was fatal in 10 patients, for a case fatality rate of 1.4%.

**Table 1.** Basic and Clinical Characteristics, Laboratory and Radiological Parameters, Hospitalization, and Case Fatality Rate Among 703 Adult Patients With Tick-Borne Encephalitis in Region Stockholm, 2006–2015

Characteristics and Parameters	Patients, No. (%)^a^ (n = 703)
Sex	
Men	430 (61.2)
Women	273 (38.8)
Age, y	
Median (range)	50 (18–94)
Mean (SD)	50 (16)
18–29	77 (11)
30–49	269 (38.3)
50–69	282 (40.1)
≥70	75 (10.7
Previous TBE vaccination	
NA	33 (4.7)
Nonvaccinated	551 (78.4)
Incompletely vaccinated	68 (9.7)
Completely vaccinated	51 (7.3)
Underlying comorbidity	
Any comorbid condition	242 (34.4)
Neurological disease	44 (6.3)
Hematological cancer	10 (1.4)
Other cancers	22 (3.1)
Inflammatory disease	43 (6.1)
Cardiovascular disease	102 (14.5)
Psychiatric disorder	58 (8.3)
Diabetes mellitus	28 (4)
Liver failure	1 (0.1)
Other	29 (4.1)
Immunomodulatory therapy	
Any	26 (3.7)
Prednisolone equivalents^b^	13 (1.9)
Methotrexate	9 (1.3)
TNF-α inhibitors	6 (0.9)
Cytostatics	4 (0.6)
Other^c^	9 (1.3)
Combination of therapies^d^	13 (1.8)
Clinical characteristic	
Biphasic course	404 (57.5)
Fever	644 (91.6)
Headache	636 (90.5)
GCS score	
Median (range)	15 (3–15)
Mean (SD)	14 (2)
Severity of disease	
Mild	313 (44.5)
Moderate	301 (42.8)
Severe	80 (11.4)
CSF findings	
Lumbar puncture performed	581 (82.7)
Pleocytosis^e^	547 (94.1)
Mononuclear lymphocyte count, cells/μL	
Median (range)	38 (0–679)
Mean (SD)	62 (75)
Polymorphonuclear leukocyte count, cells/μL	
Median (range)	6 (0–860)
Mean (SD)	20 (56)
Albumin, mg/L	
Median (range)	405 (52–1560)
Mean (SD)	441 (193)
Lactate, mmol/L	
Median (range)	2 (1–4)
Mean (SD)	2 (1)
Glucose, mmol/L	
Median (range)	3 (2–10)
Mean (SD)	3 (1)
Radiology, other diagnostics	
MR imaging	125 (17.8)
Electroencephalography	65 (9.3)
Inpatient care	
Hospitalization	529 (75.3)
Time in hospital, d	
Median (range)	7 (1–521)
Mean (SD)	13 (36)
1–7	301 (42.8)
8–14	137 (19.5)
15–29	50 (7.1)
≥30	35 (5)
ICU admission	39 (5.6)
Time in the ICU, d	
Median (range)	6 (1–519)
Mean (SD)	33 (88)
Assisted ventilation	20 (2.8)
Duration of assisted ventilation, d	
Median (range)	26 (2–609)
Mean (SD)	79 (169)
Rehabilitation	
Any rehabilitation^f^	156 (22.2)
Inpatient	88 (12.5)
Outpatient	84 (12)
Case fatalities	10 (1.4)

Abbreviations: CSF, cerebrospinal fluid; GCS, Glasgow Coma Scale; ICU, intensive care unit; MR, magnetic resonance; NA, no answer; TBE, tick-borne encephalitis; TNF, tumor necrosis factor.

^a^Data represent no. (%) of patients unless otherwise specified.

^b^Any dose of prednisolone equivalents.

^c^Including azathioprine and budesonide (3 patients each) and trastuzumab, sulfasalazine, and tacrolimus (1 patient each).

^d^Combination of ≥2 immunomodulatory therapies.

^e^Of those with lumbar puncture performed.

^f^Including inpatient care at a geriatric ward and outpatient visits to and inpatient care at a neurorehabilitation unit.

### Clinical Features in the Acute Phase and at Follow-up

Headache was the most frequently observed symptom in the acute phase (Table 2). Ataxia and dysphasia were the most common neurological signs, and mental fatigue the dominating cognitive symptom. More than half of the patients, 428 (61%), were still treated as inpatients or registered visits to the clinic between 2 weeks to 3 months after the first signs of infection. Considerably fewer patients had later inpatient treatment or follow-up visits. Among the patients with available follow-up data, cognitive disabilities were the most described persisting symptoms over the first year, with mental fatigue dominating. Ataxia and spinal nerve paresis were the most reported neurological sequelae. Of the 79 patients with a visit at 6–12 months, 71% still reported existing symptoms.

**Table 2.** Clinical Symptoms and Signs During the Acute Phase and at Follow-up Visits in Adult Patients With Tick-Borne Encephalitis in Region Stockholm, 2006–2015

Symptoms and Signs	Patients, No. (%)^a^			
	0 to <2 wk (Acute Phase) (n = 703)	≥2 wk to <3 mo (n = 428)	≥3 to <6 mo (n = 89)	≥6 to 12 mo (n = 79^b^)
Headache	636 (90.5)	142 (33.2)	25 (28.1)	18 (22.8)
Sensitivity to light/sound	215 (30.6)	32 (7.5)	11 (12.4)	5 (6.3)
Radiculitis	65 (9.3)	20 (4.7)	3 (3.4)	2 (2.5)
Cranial nerve affection	83 (11.8)	12 (2.8)	8 (9)	4 (5.1)
Spinal nerve paresis	73 (10.4)	45 (10.5)	24 (27)	18 (22.8)
Dysphasia	221 (31.4)	19 (4.4)	6 (6.7)	5 (6.3)
Apraxia	74 (10.5)	3 (0.7)	0 (0)	0 (0)
Ataxia	283 (40.3)	78 (18.2)	21 (23.6)	13 (16.5)
Epilepsy	16 (2.3)	1 (0.2)	0 (0)	0 (0)
Concentration disability	263 (37.4)	164 (38.3)	42 (42.7)	30 (38)
Memory disturbance	213 (30.3)	92 (21.5)	33 (37.1)	24 (30.4)
Mental fatigue	551 (78.4)	230 (53.7)	58 (65.2)	31 (39.2)
Emotional lability	137 (19.5)	83 (19.4)	29 (32.6)	19 (24.1)
Any of the above	700 (99.6)	315 (73.6)	73 (82)	56 (70.9)

^a^Percentages indicate the percentage of patients followed up at the specific time point.

^b^Of the 79 patients followed up at ≥6 to 12 months, 48 were also followed up at ≥3 to <6 months.

### Sex and Disease Severity

The disease severity differed between the sexes. Women were treated in the hospital to a higher extent and had longer hospital stays, but there were no significant differences between the sexes for treatment in the ICU or assisted ventilation, nor in the case fatality rate. Women participated in rehabilitation to a higher extent. The association to sex in the multivariate analysis after adjustment for age, underlying comorbid conditions, and immunomodulatory therapy, was present only for moderate disease (adjusted odds ratio [aOR], 0.7 [95% confidence interval [CI], .5–.97]) and hospitalization (0.7 [.5–.9]), using women as the reference (Supplementary Table 1).

### Underlying Comorbid Conditions, Immunomodulatory Therapy, and Age and Correlation to Outcomes

Patients with underlying comorbid conditions were older than those without comorbid conditions (Table 3). Patients with comorbid conditions were more often classified into severe or moderate disease, hospitalized, and treated in the ICU. In the multivariate analysis, using no underlying comorbidity as the reference, and after adjustment for age, sex, and immunomodulatory therapy, underlying comorbid conditions were significantly associated with severe (aOR, 3.0 [95% CI, 1.6–5.4]) and moderate (1.7 [1.2–2.5]) disease, hospitalization for >7 days (1.7 [1.2–2.6]), treatment in the ICU (2.4 [1.1–4.9]), and assisted ventilation (4.5 [1.4–14.6]). The case fatality rate was 3.3% in this group, compared with 0.4% in the group without comorbid conditions, even if no association with a fatal outcome could be shown after adjustment for age, sex, and immunomodulatory therapy (Supplementary Table 1).

**Table 3.** Clinical Characteristics in 703 Adult Patients With Tick-Borne Encephalitis in Region Stockholm, by Presence of Underlying Comorbidity, 2006–2015

Characteristic	Patients, No. (%)^a^		*P* Value
	No Comorbidity	Comorbidity	
Total	461 (65.6)	242 (34.4)	…
Age, median (range), y			
	46 (18–94)	58 (19–88)	<.001^b^
Age, mean (SD), y	46 (15)	56 (16)	
Male sex	292 (63.6)	138 (57)	.10
Clinical characteristics			
Biphasic course	286 (62)	118 (48.8)	<.001^b^
Fever	425 (92.2)	219 (90.5)	.44
Headache	426 (92.4)	210 (86.8)	.06
Severity of disease			
Mild	240 (52.1)	73 (30.2)	<.001^b^
Moderate	181 (39.3)	120 (49.6)	.009^b^
Severe	33 (7.2)	47 (19.4)	<.001^b^
Inpatient care			
Hospitalization	330 (71.6)	199 (82.2)	.001^b^
Time in hospital, d			
Median (range)	5 (1–102)	8 (1–521)	<.001^b^
Mean (SD)	8 (11)	20 (54)	
ICU admission	14 (3)	25 (10.3)	<.001^b^
Time in the ICU, d			
Median (range)	2 (1–45)	12 (1–519)	.25
Mean (SD)	10 (14)	45 (107)	
Assisted ventilation	4 (0.9)	16 (6.6)	<.001^b^
Duration of assisted ventilation, d			
Median (range)	32 (10–36)	21 (2–609)	.57
Mean (SD)	26 (14)	89 (185)	
Any rehabilitation^c^	80 (17.4)	76 (31.4)	<.001^b^
Case fatalities	2 (0.4)	8 (3.3)	.004^b^

Abbreviation: ICU, intensive care unit.

^a^Data represent no. (%) of patients unless otherwise specified.

^b^Significant at *P* < .05.

^c^Including inpatient care in a geriatric ward and outpatients visits to and inpatient care in a neurorehabilitation unit.

The same patterns were observed in patients receiving immunomodulatory therapy (Table 4). Moreover, a considerably high case fatality rate (15.4%) was observed in this group, and in the multivariate analysis, using no immunomodulatory therapy as the reference, after adjustment for age, sex, and underlying comorbid conditions, immunomodulatory therapy was significantly associated with a fatal outcome (aOR, 8. [95% CI, 1.8–36.8]; Supplementary Table 1).

**Table 4.** Clinical Characteristics in 703 Adult Patients With Tick-Borne Encephalitis in Region Stockholm, by Receipt of Immunomodulatory Therapy, 2006–2015

Characteristic	Patients, No. (%)^a^		*P* Value
	No Immunomodulatory Therapy	Immunomodulatory Therapy	
Total	677 (96.3)	26 (3.7)	…
Age, median (range), y	49 (18–94)	62 (34–83)	<.001^b^
Age, mean (SD), y	49 (16)	63 (12)	
Male sex	415 (61.3)	15 (57.7)	.71
Clinical characteristic			
Biphasic course	389 (57.5)	15 (57.7)	.98
Fever	621 (91.7)	23 (88.5)	.56
Headache	617 (91.1)	19 (73.1)	.004^b^
Severity of disease			
Mild	308 (45.5)	5 (19.2)	.008^b^
Moderate	290 (42.8)	11 (42.3)	.96
Severe	70 (10.3)	10 (38.5)	<.001^b^
Inpatient care			
Hospitalization	506 (74.7)	23 (88.5)	.04^b^
Time in hospital, d			
Median (range)	6 (1–521)	12 (2–466)	<.001^b^
Mean (SD)	11 (29)	36 (94)	
ICU admission	34 (5)	5 (19.2)	.002^b^
Time in the ICU, d			
Median (range)	5 (1–519)	27 (1–156)	.7
Mean (SD)	30 (92)	47 (62)	
Assisted ventilation	16 (2.4)	4 (15.4)	.005^b^
Duration of assisted ventilation, d			
Median (range)	25 (2–609)	26 (18–466)	.48
Mean (SD)	63 (158)	134 (221)	
Any rehabilitation^c^	144 (21.3)	12 (46.2)	.003^b^
Case fatalities	6 (0.9)	4 (15.4)	<.001^b^

Abbreviation: ICU, intensive care unit.

^a^Data represent no. (%) of patients unless otherwise specified.

^b^Significant at *P* < .05.

^c^Including inpatient care in a geriatric ward and outpatient visits to and inpatient care in a neurorehabilitation unit.

We observed a similar sex distribution in the group of patients aged ≥50 years and in the group aged <50 years (Table 5). An age ≥50 years was associated with severe and moderate disease, and these patients were more likely to be hospitalized and admitted to the ICU. In addition, hospital stays were longer in the ≥50-year age group. The case fatality rate in that group was 2.8%, whereas none in the younger age group died of the disease. In the multivariate analysis, after adjustment for sex, underlying comorbid conditions, and immunomodulatory therapy, the association remained significant for both severe (aOR, 5.8 [95%, CI, 3.1–10.8]) and moderate (1.9 [1.4–2.7]) disease in the ≥50-year age group. Moreover, hospitalization (aOR, 1.8 [95% CI, 1.2–2.5]), hospitalization >7 days (2.4 [1.7–3.5]), treatment in the ICU (3.0 [1.3–6.8]), and assisted ventilation (5.8 [1.3–25.8]) were significantly associated with older age (Supplementary Table 1).

**Table 5.** Clinical Characteristics in 703 Adult Patients With Tick-Borne Encephalitis in Region Stockholm, by Age, 2006–2015

Characteristic	Patients, No. (%)^a^		*P* Value
	Age <50 y	Age ≥50 y	
Total	346 (49.2)	357 (50.8)	…
Age, median (range)	37 (18–49)	61 (50–94)	…
Age, mean (SD)	36 (9)	62 (9)	
Male sex	214 (61.9)	216 (60.5)	.71
Clinical characteristics			
Biphasic course	237 (68.5)	167 (46.8)	<.001^b^
Fever	316 (91.3)	328 (91.9)	.79
Headache	339 (98)	297 (83.2)	<.001^b^
Severity of disease			
Mild	195 (56.4)	118 (33.1)	<.001^b^
Moderate	131 (37.9)	170 (47.6)	.009^b^
Severe	15 (4.3)	65 (18.2)	<.001^b^
Inpatient care			
Hospitalization	239 (69.1)	290 (81.2)	<.001^b^
Time in hospital, d			
Median (range)	5 (1–263)	8 (1–521)	.02^b^
Mean (SD)	8 (21)	16 (43)	
ICU admission	8 (2.3)	31 (8.7)	<.001^b^
Time in the ICU, d			
Median (range)	2 (1–79)	10 (1–519)	.47
Mean (SD)	12 (27)	38 (98)	
Assisted ventilation	2 (0.6)	18 (5)	<.001^b^
Duration of assisted ventilation, d			
Median (range)	25 (4–46)	26 (2–609)	.65
Mean (SD)	25 (30)	85 (179)	
Any rehabilitation^c^	47 (13.6)	109 (30.5)	<.001^b^
Case fatalities	0	10 (2.8)	…

Abbreviation: ICU, intensive care unit.

^a^Data represent no. (%) of patients unless otherwise specified.

^b^Significant at *P* < .05.

^c^Including inpatient care in a geriatric ward and outpatient visits to and inpatient care in a neurorehabilitation unit.

### TBE Vaccination Status and Disease Severity

A subanalysis was performed on the 670 patients who had accessible data regarding previous TBE vaccination (Table 6). The majority were nonvaccinated (82%), 68 patients (10%) were vaccinated but had not adhered to the recommended vaccination schedule (incompletely vaccinated), while 51 (8%) were previously completely vaccinated. Data from the completely vaccinated subgroup have been thoroughly described elsewhere [17]. Within the incompletely vaccinated group, 31 individuals had received only 1 previous TBE vaccine dose, and 18 had received 2 doses more than a year before onset of disease (data not shown).

**Table 6.** Basic and Clinical Characteristics, Laboratory and Radiological Parameters, Hospitalization, and Mortality Rate in 670 Adult Patients With Tick-Borne Encephalitis (TBE) in Region Stockholm by TBE Vaccination Status, 2006–2015

Variable	Patients, No. (%)^a^			*P* Value		
	NV	IV	CV	NV vs CV	IV vs CV	NV vs IV
Total	551 (82.2)	68 (10.1)	51 (7.6)	…	…	…
Sex						
Male	339 (61.5)	34 (50)	32 (62.8)	.86	.17	.07
Female	212 (38.5)	34 (50)	19 (37.3)			
Age, median (range), y	48 (18–94)	55 (21–86)	62 (19–83)	<.001^b^	.04^b^	.002^b^
Age, mean (SD), y	48 (15)	54 (17)	60 (14)			
Any underlying comorbidity	175 (31.8)	26 (38.2)	29 (56.9)	<.001^b^	.04^b^	.28
Immunomodulatory therapy						
Any	14 (2.5)	3 (4.4)	7 (13.7)	.001^b^	.09	.42
Prednisolone equivalents^c^	6 (1.1)	3 (4.4)	2 (3.9)	.14	.89	.06
Methotrexate	4 (0.7)	1 (1.5)	4 (7.8)	.003^b^	.16	.44
TNF-α inhibitors	3 (0.5)	1 (1.5)	2 (3.9)	.06	.57	.37
Cytostatics	3 (0.5)	0 (0)	0 (0)	.59	…	.54
Other	7 (1.3)	0 (0)	1 (2)	.51	.42	.44
Combination of therapies^d^	8 (1.5)	2 (2.9)	2 (3.9)	.18	1	.3
Clinical characteristics						
Biphasic course	351 (63.7)	25 (36.8)	12 (23.3)	<.001^b^	.12	<.001^b^
Fever	500 (90.7)	65 (95.6)	48 (94.1)	.60	.72	.18
Headache	508 (92.2)	59 (86.8)	41 (80.4)	.17	.86	.03
Severity of disease						
Mild	261 (47.4)	26 (38.2)	10 (19.6)	<.001^b^	.03^b^	.15
Moderate	237 (43)	32 (47.1)	20 (39.2)	.59	.39	.52
Severe	46 (8.4)	10 (14.7)	20 (39.2)	<.001^b^	.002^b^	.08
CSF findings						
Lumbar puncture performed	449 (81.5)	60 (88.2)	49 (96.1)	.008^b^	.18	.17
Pleocytosis^e^	425 (77.1)	54 (79.4)	49 (96.1)	.15	.03^b^	.15
Albumin, mg/L						
Median (range)	400 (131–1560)	415 (136–1440)	529 (254–1198)	.009^b^	.5	.1
Mean (SD)	507 (160)	479 (255)	430 (183)			
Radiology, other diagnostics						
MR imaging	82 (14.9)	14 (20.6)	24 (47.1)	<.001^b^	.002^b^	.22
Electroencephalography	32 (5.8)	10 (14.7)	21 (41.2)	<.001^b^	.001^b^	.02^b^
Inpatient care						
Hospitalization	403 (73.1)	52 (76.5)	44 (86.3)	.02^b^	.1	.54
Time in hospital, d						
Median (range)	6 (1–102)	8 (1–93)	14 (1–521)	<.001^b^	.05^b^	.005^b^
Mean (SD)	9 (11)	14 (18)	52 (102)			
ICU admission	15 (2.7)	9 (13.2)	12 (23.5)	<.001^b^	.14	<.001^b^
Time in the ICU, d						
Median (range)	2 (1–45)	17 (1–78)	7 (1–519)	.2	.38	.11
Mean (SD)	9 (14)	23 (25)	70 (155)			
Assisted ventilation	4 (0.7)	5 (7.4)	8 (15.7)	<.001^b^	.15	<.001^b^
Duration of assisted ventilation, d						
Median (range)	21 (4–35)	34 (6–40)	21 (2–609)	.3	.33	.48
Mean (SD)	20 (16)	29 (16)	165 (258)			
Case fatalities	2 (0.4)	4 (5.9)	3 (5.9)	.005^b^	…	.002^b^

Abbreviations: CSF, cerebrospinal fluid; CV, completely vaccinated; ICU, intensive care unit; IV, incompletely vaccinated; NV, nonvaccinated; TNF, tumor necrosis factor;

^a^Data represent no. (%) of patients unless otherwise specified.

^b^Significant at *P* < .05.

^c^Any dose of prednisolone equivalents.

^d^Combination of ≥2 immunomodulatory therapies.

^e^Of those with lumbar puncture performed.

Completely vaccinated patients, compared with the nonvaccinated group, were older and had a higher prevalence of underlying comorbid conditions and immunomodulatory therapy. In the completely vaccinated group, a higher proportion of severe infection was observed, as well as higher rates of hospitalization, ICU admission, and assisted ventilation and longer hospital stays. In patients with previous vaccinations (both incompletely and completely vaccinated) a biphasic course was less often observed, and these patients were more often treated in the ICU and had a higher case fatality rate (5.9%). Multivariate analysis was performed using nonvaccinated patients as the reference, with adjustment for age, sex, underlying comorbid conditions, and immunomodulatory therapy. Severe disease (aOR, 6.2 [95% CI, 2.4–16.0]), hospitalization >7 days (4.2 [1.8–10.2]), treatment in the ICU (6.6 [2.7–15.9]), and assisted ventilation (12.2 [3.3–44.7]) were significantly associated with being completely vaccinated. Significant association with incomplete vaccination status in the multivariate analysis, still using nonvaccinated patients as the reference, was seen for hospitalization >7 days (aOR, 2.1 [95% CI, 1.1–4.1]), treatment in the ICU (4.5 [1.8–11.3]), assisted ventilation (8.8 [2.1–36.5]), and fatal outcome (12.6 [2.0–81.3]; Supplementary Table 1).

## DISCUSSION

TBE is an emerging infectious disease of substantial public health importance, associated with significant morbidity and mortality rates. This is the largest cohort of patients with TBE studied in Sweden. The majority of the patients were previously nonvaccinated, and more cases occurred in men, similarly to findings in other European reports [6, 7, 9]. Fever, headache, and fatigue was the most noted clinical characteristics in the acute phase of disease. Ataxia was the most frequently observed neurological symptom, exceeding what has been previously observed [20, 25].

Many patients with TBE require hospitalization [9]. A recent study from Germany reported a hospitalization rate of almost 90%, higher than the 75% that we observed [26]. The length of hospital stays vary and has been reported at a median between 8–23 days, slightly longer than in our study [7, 26–29]. A median hospitalization of 7 days is comparable to that in previous Swedish cohorts [20, 21]. A significant proportion of patients with TBE are also treated within the intensive care unit (ICU), ranging from 7% to 30%, whereas we noted ICU treatment in about 6% of our cohort [7, 8, 26, 27, 29]. Difficulties in comparing hospitalization rates and lengths of stay between different countries include traditions and recommendations for hospitalizations as well as the number of hospital beds, where Sweden, together with 5 other European Union countries, has the lowest number of beds relative to population size [30].

There are also differences regarding the number of ICU beds and definitions of intensive care. The proportion of patients treated with assisted ventilation in our cohort (3%), is similar to that in other reports [7, 8, 29], potentially making this outcome easier to compare between different hospital settings. There are only a few studies on rehabilitation after TBE. In our cohort, about a fifth of the patients underwent any rehabilitation, similar to the rate in a German study from 1999 [31] but lower than what has been described more recently [26, 32]. Further research is needed on rehabilitation of patients with TBE to understand what best benefits these patients.

A variety of definitions on disease severity is used in studies on TBE. We used a similar classification of severity as used in a prospective study in Lithuania, and we demonstrated a distribution of severe and moderate disease consistent with the findings of that study, about 10% severe and 40% moderate cases [11]. Moreover, the distribution of severity was quite similar to that in previous Swedish studies, [20, 25]. Other studies categorize patients according to their clinical form of disease, where meningitis is comparable to mild disease in the classification used in our study, meningoencephalitis to moderate or severe disease, and meningoencephalomyelitis to severe disease, with distributions ranging from 36%–58% for meningitis, 28%–60% for meningoencephalitis, and 4%–14% for meningoencephalomyelitis [7, 28, 29, 33].

Increasing age has been demonstrated to be correlated with disease severity [8, 11, 26–29, 31, 33]. Here, we found that patients aged ≥50 years were more likely to be hospitalized, hospitalized for a longer period, and admitted to the ICU, as well as treated with assisted ventilation. Of note, there were no deaths among patients <50 years of age. The overall case fatality rate was 1.4%, in accordance with prior reports from Europe and Sweden [6–9, 21, 34]. We found far from negligible case fatality rates in patients ≥50 years old (2.8%), those with underlying comorbid conditions (3.3%), and especially in those receiving immunomodulatory therapy (15.4%). In addition to the higher case fatality rate among patients with underlying comorbid conditions, they had more severe disease, were hospitalized, and were treated in the ICU, including assisted ventilation, to a greater extent than patients without comorbid conditions.

Comorbid conditions have previously been found to be one of the prognostic factors associated with severe disease and severe meningoencephalitis [8]. A similar pattern of more severe disease and increased healthcare utilization was observed in patients receiving immunomodulatory therapy; however, the considerably high rate of fatal outcomes in this group is particularly noteworthy. Case fatality was also the only variable associated with immunomodulatory treatment in the multivariate analysis. To our knowledge, this is the first study on the outcomes of TBE in patients receiving immunomodulatory therapy, even if multiple case reports have shown fatal TBE infection in immunosuppressed patients [35–37]. Our findings are implicative of a need for heightened vigilance in patients with TBE receiving immunomodulatory therapy and in those with comorbid conditions, as well as in those who are older.

Of the 10 patients who died, 8 were men, but the association with sex was present only for moderate disease and hospitalization. Similarly, in prior studies, disease severity did not differ by sex [26], nor was it associated with any specific clinical diagnoses [7]. In contrast, one study demonstrated that male sex was a risk factor for meningoencephalitis and meningoencephaloradiculatis [33]. Hence, there are somewhat conflicting results regarding a more severe outcome in men, and it remains unclear why men are affected by TBE to a greater extent than women.

We aimed to collect data for up to 12 months after onset of disease, but only 11% of the patients in our cohort had follow-up visits at the hospitals after 6 months. A high rate of the patients who were followed up reported persisting symptoms, mostly cognitive disturbances and headache, but also ataxia and spinal nerve paresis, similarly to reports in previous studies, although the frequency of long-term symptoms in our cohort is somewhat higher than what has been previously described [7, 10, 11, 21, 25, 31, 38, 39]. These findings could indicate the bias of including only hospital-based visits. In addition, the dropout of patients throughout the follow-up period may have had an increasing effect on the rates of sequelae, as it is likely that patients with persisting symptoms have further visits planned and may be more willing to adhere to them.

Vaccination breakthrough infection has been described in older patients [17, 40] and with a severe course of disease [13, 17, 27, 40], whereas no differences in age and disease severity have been found in other studies [7, 41]. In our study, a total of 119 patients had been previously vaccinated against TBE, of whom 51 had adhered to the recommended vaccination schedule. The previously completely vaccinated patients were older, had more comorbid conditions and immunomodulatory medications, had more severe disease, and were hospitalized to a greater extent and for longer, compared with both previously nonvaccinated and incompletely vaccinated patients. Strikingly, both completely and incompletely vaccinated patients, compared with nonvaccinated patients, were admitted to the ICU and treated with assisted ventilation to a greater extent, together with having a higher case fatality rate. With increased numbers of people vaccinated against the disease, an increasing number of vaccination breakthrough infections will be diagnosed, and it is thus important to understand these infections.

Importantly, our study was not designed to identify all breakthrough infections that occur, as we identified only patients seeking hospital care. As for our cohort, there is also most likely a diagnostic bias in the vaccination breakthrough population, as this group is more difficult to diagnose given that the current cornerstone for diagnosis of TBE is based on serology [42]. Patients with less severe breakthrough infection would thus not be diagnosed with TBE. In addition, the declining priming vaccination response in older and immunosuppressed individuals will inevitably lead to more breakthrough infections in these groups, as with other vaccines and diseases [43–45]. Given the selected cohort, our findings cannot find associations between vaccination per se and worsened outcome but rather underline the importance of initializing TBE immunization early in life, for optimal priming and before immunosenescence due to older age or potential immunomodulatory treatment.

Recent data on TBE virus (TBEV) infection and vaccination rates in Region Stockholm, based on serological assessment in blood donors, suggest that 874 500 people (57%) in Region Stockholm are vaccinated against the disease and 107 400 (7%) have had the infection, as assessed with whole-virus and NS1 multiplex assay [46], leaving the risk of clinically detectable vaccination breakthrough infections to very low levels, which would be in accordance with the high real-world effectiveness of available TBE vaccines [47]. Similar data on high incidence of predominantly silent TBEV infections (5.6% TBEV NS1 IgG seroprevalence) have been described in southwestern Germany [48].

This study has several limitations, including the retrospective design based on the assessment of medical records and the fact that patients included in the study were all examined at and/or treated in a hospital, excluding patients with milder disease who were handled at primary care centers. Consequently, only a selection of the TBEV-infected, especially patients who were more severely ill, was included in this study. Likewise, concerning sequelae, since we studied patients only with follow-up visits at a hospital we may have observed only the more severely affected patients with TBE.

A main strength of the current study is that the mandatory notifying system for TBE cases, with retrieval of medical records for 703 of the 704 reported cases, allowed for sound data collection and analysis. We also had access to detailed information on vaccinations for the majority of the patients.

In conclusion, TBE is associated with a high morbidity rate and risk of sequelae. The overall case fatality rate is relatively low but is considerable in older patients, in patients with underlying comorbid conditions, and especially in patients receiving immunomodulatory therapy. Severe TBE is associated with age ≥50 years, with underlying comorbid conditions, and with vaccination breakthrough infection. These findings can contribute to clinical practice and the care of patients with TBE and provide a base for the further assessment needed to understand the mechanisms of severe disease.

## Supplementary Data

Supplementary materials are available at *The Journal of Infectious Diseases* online (http://jid.oxfordjournals.org/). Supplementary materials consist of data provided by the author that are published to benefit the reader. The posted materials are not copyedited. The contents of all supplementary data are the sole responsibility of the authors. Questions or messages regarding errors should be addressed to the author.

## Supplementary Material

jiae463_Supplementary_Data

## Notes

***Author contributions.*** S. B., M-P. H., H. H. A., and S. G.-R. conceived and designed the study. S. B., M-P. H., K. E. H., J. R., P. H., I. K., M. I., H. H. A., and S. G.-R. were responsible for data collection, analysis, and interpretation of data. S. B. and S. G.-R. wrote the manuscript, with support from M-P. H., K. E. H., J. R., P. H., I. K., M. I., S. V., L. L., and H. H. A. All authors approved the manuscript for publication.

***Financial support*.** This work was supported by Region Stockholm (ALF project and Clinical Research Appointment); the Center for Innovative Medicine, Region Stockholm; the Swedish Research Council (Diary Number 2020-06249 and 2021-06602); and the Marianne and Marcus Wallenberg Foundation (all to S. G.-R.).
