# Supplementary material for: Clinical Characteristics of Tick-Borne Encephalitis in Adult Patients: A 10-year Retrospective Study in Stockholm, Sweden
Source: J Infect Dis. 2024 Sep 24;231(1):e195–205. doi: 10.1093/infdis/jiae463 (PMC11793045; doi:10.1093/infdis/jiae463)
Supplement: jiae463_Supplementary_Data [file jiae463_supplementary_data.docx]

| **Supplementary Table 1. Adjusted OR with 95% CI for Several Predefined Outcomes for Disease Severity in Adult Patients with Tick-borne encephalitis, in Region Stockholm during 2006 to 2015.** | | | | | | | |
| --- | --- | --- | --- | --- | --- | --- | --- |
|  | Moderate disease | Severe disease | Hospitalis-ation | Hospitalis-ation >7 days | Treatment in the ICU | Assisted ventilation | Case fatality |
|  |  |  |  |  |  |  |  |
|  | aOR (95% CI) | aOR (95% CI) | aOR (95% CI) | aOR (95% CI) | aOR (95% CI) | aOR (95% CI) | aOR (95% CI) |
| Women | Reference |  |  |  |  |  |  |
| Men | 0.7 (0.5-0.97) | 1.6 (0.9-3.1) | 0.7 (0.5-0.9) | 0.7 (0.5-1.1) | 1.3 (0.7-2.7) | 2.1 (0.7-6.1) | 2.8 (0.6-14.1) |
| No underlying comorbities | Reference |  |  |  |  |  |  |
| Comorbidities | 1.7 (1.2-2.5) | 3.0 (1.6-5.4) | 1.4 (0.9-2.1) | 1.7 (1.2-2.6) | 2.4 (1.1-4.9) | 4.5 (1.4-14.6) | 2.4 (0.4-13.5) |
| No immunomodulatory therapy | Reference |  |  |  |  |  |  |
| Immunomodulatory therapy | 1.2 (0.4-3.5) | 2.8 (0.8-9.5) | 2.4 (0.5-2.1) | 2.4 (0.8-7.6) | 2.1 (0.7-6.4) | 2.7 (0.8-9.4) | 8.2 (1.8-36.8) |
| Age <50 years | Reference |  |  |  |  |  |  |
| Age ≥50 years | 1.9 (1.4-2.7) | 5.8 (3.1-10.8) | 1.8 (1.2-2.5) | 2.4 (1.7-3.5) | 3.0 (1.3-6.8) | 5.8 (1.3-25.8) | - * |
| Non-vaccinated | Reference |  |  |  |  |  |  |
| Completely vaccinated | 1.6 (0.7-3.6) | 6.2 (2.4-16.0) | 1.8 (0.7-4.4) | 4.2 (1.8-10.2) | 6.6 (2.7-15.9) | 12.2 (3.3-44.7) | 6.1 (0.95-38.8) |
| Non-vaccinated | Reference |  |  |  |  |  |  |
| Incompletely vaccinated | 1.2 (0.7-2.1) | 1.5 (0.6-3.7) | 1.0 (0.5-1.8) | 2.1 (1.1-4.1) | 4.5 (1.8-11.3) | 8.8 (2.1-36.5) | 12.6 (2.0-81.3) |
| *No patient <50 years of age died | | | | |  |  |  |
